# Rosiglitazone Improves Glucocorticoid Resistance in a Sudden Sensorineural Hearing Loss by Promoting MAP Kinase Phosphatase-1 Expression

**DOI:** 10.1155/2019/7915730

**Published:** 2019-05-14

**Authors:** Liang Xia, Jingjing Liu, Yuanyuan Sun, Haibo Shi, Guang Yang, Yanmei Feng, Shankai Yin

**Affiliations:** ^1^Department of Otolaryngology, Shanghai Jiao Tong University Affiliated Sixth People's Hospital, No. 600, Yishan Road, Xuhui District, 200233 Shanghai, China; ^2^Department of Interventional Radiology, Shanghai Jiao Tong University Affiliated Sixth People's Hospital, No. 600, Yishan Road, Xuhui District, 200233 Shanghai, China

## Abstract

In this study, we investigated the role of MAP kinase phosphatase-1 (MKP-1) and rosiglitazone (RSG) in glucocorticoid resistance and glucocorticoid sensitivity, respectively, using a guinea pig model of lipopolysaccharide- (LPS-) induced sudden sensorineural hearing loss (SSHL). The pigs were divided into control, LPS, LPS+dexamethasone (DEX), LPS+RSG, and LPS+DEX+RSG groups. Their hearing was screened by auditory brainstem response measurement. Immunofluorescence staining was used to identify the location of MKP-1 in the inner ear. The expression levels of MKP-1 and the related proteins in the inner ear were detected using western blotting. The morphological changes in the cochlea were observed via hematoxylin-eosin staining. Severe hearing loss was observed in the LPS group, as opposed to the protection from hearing loss observed in the LPS+DEX+RSG group. A positive correlation was observed between MKP-1 expression levels and protection from hearing loss. RSG and DEX synergistically influenced inner ear inflammation. In conclusion, resistance of LPS-induced SSHL guinea pig models to glucocorticoids may result from impaired MKP-1 function in inner ear tissues, induced by glucocorticoids, impairing the inhibition of inflammation. Our findings present novel targets to develop potential therapeutics to treat inflammatory diseases of the inner ear.

## 1. Introduction

An increasing number of studies have reported that inflammation and oxidative stress may lead to sudden sensorineural hearing loss (SSHL) and affect prognosis [1–3]. Glucocorticoids are the main treatment option for SSHL. They play a major role in maintaining homeostasis, including immune function regulation. Dexamethasone (DEX), a synthetic glucocorticoid, has been widely used for the treatment of inner ear disorders such as SSHL, Ménière disease, and acute tinnitus. Although recent clinical studies have shown that glucocorticoid therapy is effective against inner ear diseases, a considerable number of patients are insensitive and thus resistant to glucocorticoids. Thus, there is an urgent need for effective drugs that prevent disease progression. Proinflammatory cytokines and other mediators are presumed to contribute to the development of glucocorticoid insensitivity or resistance. For instance, reduced expression of glucocorticoid receptor (GR) and histone deacetylase-2 (HDAC2) leads to glucocorticoid insensitivity or resistance [4, 5]. A recent study suggested that the activity of mitogen-activated protein kinase (MAPK) phosphatase 1 (MKP-1: NCBI official full name, dual-specificity phosphatase 1 (DUSP1)) is related to corticosteroid insensitivity or resistance [6].

MKPs belong to the family of DUSPs, which play a role in dephosphorylating their substrates [7–9]. The MAPK family comprises of three stress-activated protein kinase pathways: p38, c-Jun N-terminal kinase (JNK), and extracellular regulating kinase (ERK) [10]. The ERK pathway is mainly activated by mitogenic and proliferative stimuli, while the p38 MAPK and JNK pathways respond to environmental stresses [11]. MKP-1 is a protein that exerts anti-inflammatory function by efficaciously dephosphorylating the JNK and p38 MAPK pathways and deactivating the nuclear factor-*κ*B (NF-*κ*B) pathway in immune cells [12–14]. In addition, MKP-1 plays a critical role in controlling the extent and duration of proinflammatory MAPK signaling *in vivo*. However, alveolar macrophages from patients with severe asthma (glucocorticoid-resistant) showed reduced induction of MKP-1 expression, due to the activation of p38 MAPK [15]. In patients with chronic obstructive pulmonary disease and patients who smoke, MKP-1 may be inactive owing to its oxidation, which may contribute to glucocorticoid resistance [6]. However, the role of MKP-1 in the cochleae has not been reported.

Rosiglitazone (RSG), a peroxisome proliferator-activated receptor gamma (PPAR-*γ*) agonist, was reported to induce MKP-1 expression [16]. Another study showed that RSG can potentially be used as a therapeutic agent for acute inflammatory conditions and acute liver injury [17].

In this, as in other studies, a guinea pig model of SSHL was induced by intracochlear injection of lipopolysaccharides (LPS) [5, 18, 19]. Using this model, we demonstrate for the first time how MKP-1 protects inner ear tissue against inflammation-induced morphological damage and dysfunction. We also elucidated the synergistic effect of RSG and DEX on MKP-1 expression and glucocorticoid resistance. Therefore, this study may offer novel targets for potential therapeutics for treating inflammatory diseases of the inner ear.

## 2. Materials and Methods

### 2.1. Drugs

LPS and RSG were purchased from Sigma-Aldrich Chemical Co. (St. Louis, USA). DEX was purchased from Shanghai Jiao Tong University Affiliated Sixth People's Hospital (Shanghai, China).

### 2.2. Ethics and Animals

All animal experiments were approved by the Ethics Committee of the Shanghai Jiao Tong University Affiliated Sixth People's Hospital. And the care and handling of guinea pigs were approved by the Institutional Animal Care and Use Committee and preceded in accordance with the Animals (Scientific Procedures) Act 1986 (amended 2013) [20]. All guinea pigs were housed in individually ventilated cages (two or three per cage) under specific pathogen-free (SPF) conditions.

### 2.3. Animal Models and Drug Treatments

Thirty male albino guinea pigs with an average body weight of 250 g were randomly divided into five groups (*n* = 6) including the AP (artificial perilymph), LPS, LPS+DEX, LPS+RSG, and LPS+DEX+RSG groups. AP cochlear perfusion was performed on the pigs in the AP group. LPS cochlear perfusion was performed on the pigs in the LPS group. LPS cochlear perfusion performed on and DEX was intraperitoneally injected into the pigs in the LPS+DEX group. LPS cochlear perfusion was performed on and RSG was intraperitoneally injected into the pigs in the LPS+RSG group. LPS cochlear perfusion was performed on and DEX and RSG were intraperitoneally injected into the pigs in the LPS+DEX+RSG group. DEX (1 mg/kg) or RSG (3 mg/kg, diluted in dimethyl sulfoxide) or both were intraperitoneally injected 30 min before surgery and 24 h after surgery. The subjects were placed in a heating pad with thermostatic control to maintain their body temperature at 38°C. Cochleostomy was performed on inhalant isoflurane-anesthetized pigs (4% for induction, 2% for maintenance, and 0.3 L/min O_2_ flow rate) for injecting LPS (5 mg/mL) or AP (NaCl 145 mM, KCl 2.7 mM, MgSO_4_ 2.0 mM, CaCl_2_ 1.2 mM, and HEPES, C_8_H_18_N_2_O_4_S 5.0 mM). Lidocaine (1%) was subcutaneously administered in their postauricular regions. The posterior part of their auditory bulla was bluntly dissected. Holes, 0.3 mm in diameter, were punctured into their mastoid bulla to expose the basal turn of the cochlea. The holes were accessed through the bony wall of the scala tympani of the basal turn in the cochlea. Cochlear injections were administered using a glass tip made from a 34 G microfilm, connected to a microsyringe pump (Micro4; WPI, Kissimmee, USA) through a polyethylene tube. Then, 5 *μ*L of LPS/AP was injected at a rate of 50 nL/s into the scala tympani. The cochleostomy holes were closed with muscle tissue, while the holes made in their bulla were closed with muscle suture and skin incision.

### 2.4. Auditory Brainstem Response (ABR) Tests

ABR thresholds were determined before surgery and 48 h after surgery in all subjects. The mean threshold shifts were averaged from two ears in all subjects. First, the guinea pigs were anesthetized with ketamine (40 mg/kg) mixed with xylazine (4 mg/kg, ip). Body temperature was maintained at 38°C. For closed field ABR tests, sound signal was passed through a plastic tube to the tested ear. Three subcutaneous electrodes were used to record the reaction. The recording electrode was inserted at the vertex, while the reference and grounding electrodes were inserted behind the external auditory canal. The ends of the three electrodes were connected to a RA16PA preamplifier. TDT System III (Tucker-Davis Technologies, Alachua, FL, USA) was used for stimuli generation. The stimuli were played through a broadband speaker (MF1; TDT). The sound level was decreased by 5 dB steps from 90 dB SPL until the response disappeared. The ABR thresholds were tested at 1, 2, 4, 8, 16, and 32 kHz, which are the lowest levels at which repeatable wave III responses can be recorded.

### 2.5. Immunofluorescence

To investigate the expression of MKP-1 in the cochlea of guinea pigs, the cochlea was removed after the ABR tests. The cochlea was fixed in 4% paraformaldehyde in phosphate-buffered saline (PBS) for 2 h at 4°C. Then, the cochlea was transferred into PBS and its bony shell was removed. After removing the tectorial membrane, the basilar membrane and spiral ganglion (SGN) were carefully peeled off, followed by immersion in PBS containing 1% Triton X-100 for 1 h and incubation in 5% goat serum for 1 h. Next, the basilar membrane and spiral ganglion were incubated with primary rabbit anti-MKP-1 antibody (1 : 100) (Affinity Biosciences, USA) for 20 h at 4°C. This was followed by incubation with the Alexa Fluor® 488 goat anti-rabbit IgG (secondary antibody) (1 : 500) (Abcam, Cambridge, UK) for 2 h at 25°C. Fluorescein isothiocyanate- (FITC-) phalloidin (1 : 500) (Cytoskeleton Inc., CO, USA) was used to stain hair cell stereocilia bundles. 4′,6-Diamidino-2-phenylindole (DAPI) (Sigma-Aldrich, USA) was used for nucleic acid staining. The sections were cover-slipped, examined with the LSM 710 confocal microscope (Zeiss, Oberkochen, Germany), and analyzed by the ZEN 2011 software (Zeiss, Oberkochen, Germany).

### 2.6. Hematoxylin-Eosin Staining

Pathological changes in the cochlea of guinea pigs were observed using hematoxylin-eosin (HE) staining. The cochlea of pigs in all groups was decalcified in ethylenediaminetetraacetic acid (EDTA) and then dehydrated in ethanol. They were then imbedded in paraffin. Thereafter, they were sectioned to a 3 *μ*m thickness and stained with HE. The sections were observed with the LSM 710 META confocal laser scanning microscope (Zeiss, Shanghai, China). SGN cells from base to the apex in each section of Rosenthal's canal were counted. Lastly, SGN cell density (number/10000 *μ*m^2^) was analyzed.

### 2.7. Western Blot Analysis

Extracted proteins from cochlea samples were prepared in radioimmunoprecipitation assay (RIPA) buffer mixed with the protease inhibitor phenylmethanesulfonyl fluoride (PMSF) at a 100 : 1 ratio (Beyotime Institute of Biotechnology, Shanghai, China). The protein concentrations were detected using the Bicinchoninic Acid (BCA) Protein Assay Kit (Beyotime Institute of Biotechnology). Sample lysates (30 *μ*g protein per lane) were then subjected to 10% sodium dodecyl sulfate–polyacrylamide gel electrophoresis (SDS-PAGE) and transferred to polyvinylidene difluoride (PVDF) membranes with a pore size of 0.22 *μ*m (Millipore, Billerica, MA, USA). The membranes were blocked for 2 h at room temperature with 5% nonfat milk in Tris-buffered saline with Tween-20 (TBST) and then incubated with the appropriate primary antibodies (1 : 800–1 : 1000 dilution) at 4°C for 20 h. After washing three times for 10 min with TBST, the membranes were incubated with HRP-conjugated secondary antibodies (1 : 5000, Proteintech, Chicago, IL, USA) for 1 h at 25°C. The antibodies binding to the blots were visualized on a GE Amersham Imager 600 imaging system using an enhanced chemiluminescence detection kit (Cell Signaling Technology, Boston, MA, USA). The results are expressed as a percentage of GAPDH to express relative protein levels. Antibodies to p38 MAPK (cat. no 8690S), phospho-p38 MAPK (cat. no. 4511S), NF-*κ*B p65 (cat. no. 8242S), phospho-NF-*κ*B p65 (cat. no. 3033S), and GAPDH (cat. no. 5174S) were purchased from Cell Signaling Technology. Anti-MKP-1 (cat. no. AF5286) was purchased from Affinity Biosciences (Cincinnati, OH, USA). Anti-GR (cat. no. ab3578) was purchased from Abcam.

### 2.8. Statistical Analysis

All statistical analyses were performed using SPSS version 23.0 (IBM Corp., Armonk, NY, USA). Descriptive statistics were calculated for each group. One-way analyses of variance (ANOVA) was used to assess the differences between groups. The significance level was set at *p* < 0.05.

## 3. Results

### 3.1. Evaluation of Hearing Function in Each Group

**Figure 1:**
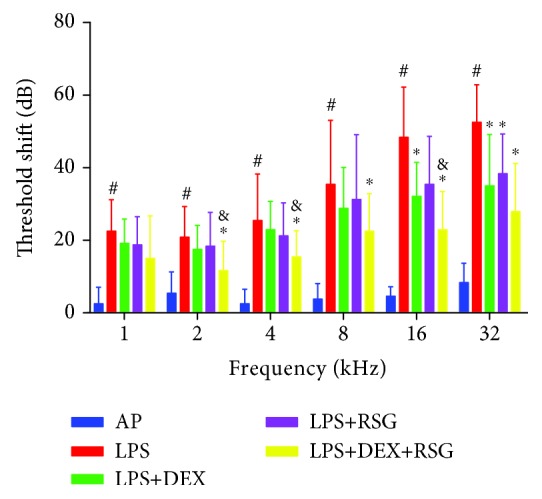
Hearing threshold shifts in each group were measured using the ABR tests before and 48 h after surgery at different frequencies. ^#^*p* < 0.05 vs. AP group; ^∗^*p* < 0.05 vs. LPS group; and *p* < 0.05 vs. LPS+DEX group.

The hearing threshold shift in each group was measured using the ABR tests before and 48 h after surgery at different frequencies (Figure 1). The average threshold shift at each frequency (1, 2, 4, 8, 16, and 32 kHz) in the AP group was less than 10 dB, indicating the surgery had no effect on hearing function. The threshold shift in the LPS group was significantly higher than that in the AP group (*p* < 0.05). Also, the extent of hearing impairment increased with frequency. The threshold shift in the LPS+DEX group decreased compared to that in the LPS group; however, significant differences were observed only at 16 and 32 kHz. This suggests that DEX has only a partial effect on LPS-induced hearing loss and glucocorticoid resistance. The threshold shift in the LPS+RSG group decreased partially, but there was no significant difference when compared with that of the LPS group, except at 32 kHz. The LPS+DEX+RSG group possessed a significant hearing protection. The threshold shift decreased significantly compared to that in the LPS group at 2, 4, 8, 16, and 32 kHz. Moreover, the threshold shift decreased significantly at 2, 4, and 16 kHz for the LPS+DEX+RSG and LPS+DEX groups. These findings show that LPS causes a significant hearing loss in the inner ear, and the combination of RSG and DEX substantially protects against hearing loss.

### 3.2. Immunostaining of MKP-1 in the Basilar Membrane and Spiral Ganglion

**Figure 2:**
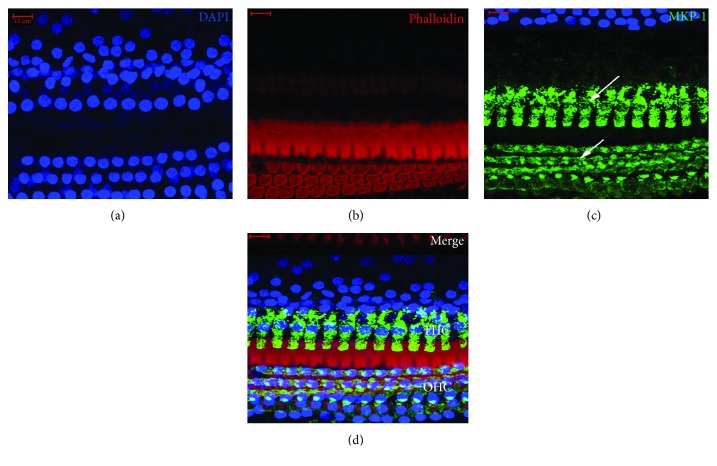
MKP-1 immunofluorescence staining of cochlear basilar membranes of pigs in the AP group. MKP-1 stained in the spiral ganglion cell (white arrows). Blue: nuclei stained with DAPI; red: stereocilia stained with FITC-phalloidin; green: MKP-1 stained in inner hair cell (IHC) and outer hair cell (OHC). Scale bars = 10 *μ*m.

**Figure 3:**
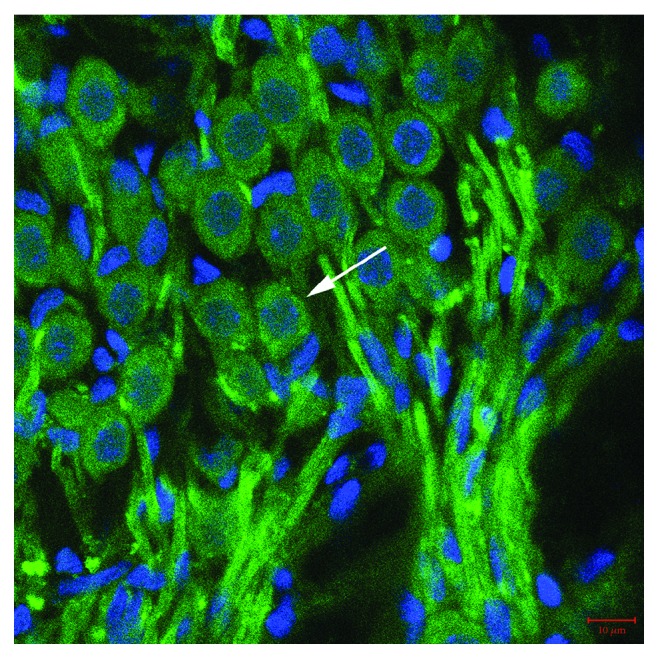
MKP-1 immunofluorescence staining of cochlear spiral ganglion of pigs in the AP group. MKP-1 stained in the spiral ganglion cell (white arrows). Blue: nuclei stained with DAPI; green: MKP-1 stained in inner hair cell (IHC) and outer hair cell (OHC). Scale bars = 10 *μ*m. The red marker is the stereocilia of hair cells, and the blue marker is the cell nucleus.

**Figure 4:**
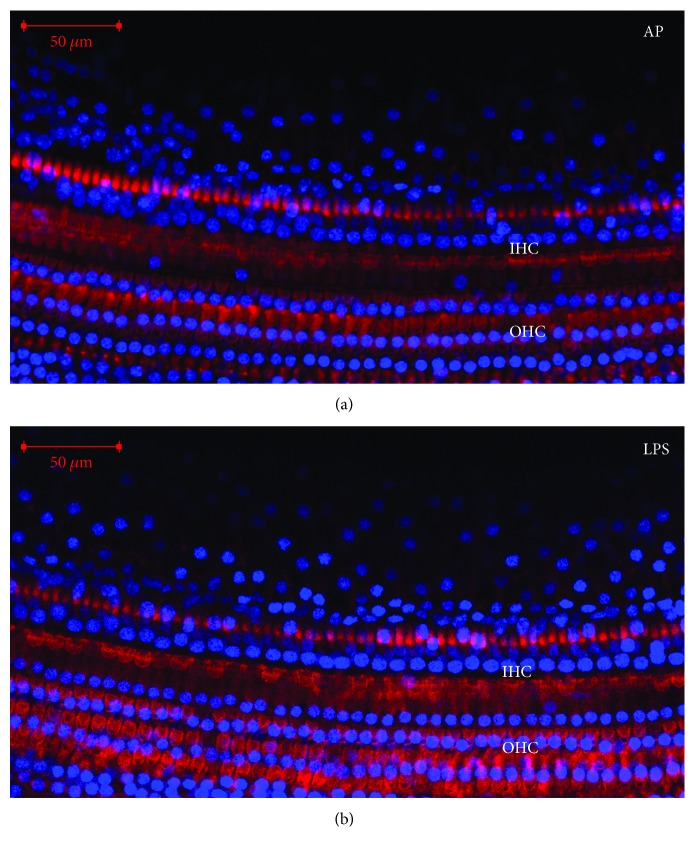
The immunostaining of basilar membranes of pigs in the AP and LPS groups. Blue: nuclei stained with DAPI; red: stereocilia stained with FITC-phalloidin. Scale bars = 50 *μ*m. Compared to the AP group, the LPS group showed no obvious loss of inner and outer hair cells, and the structure of stereocilia was normal.

As shown in Figure 2, MKP-1 immunofluorescence staining of cochlear basilar membranes in the AP group showed MKP-1 is located in the hair cell nucleus and cytoplasm. Also, Figure 3 shows the localization of MKP-1 in the spiral ganglion of pigs in the AP group. However, MKP-1 is abundant in the cytoplasm of neurons than in hair cells. There was no significant difference in the location of MKP-1 among different groups.  Figure 4 shows the immunostaining of complete basilar membranes in the AP and LPS groups. Compared to the AP group, the LPS group showed no obvious loss of inner or outer hair cells, and the structure of stereocilia was normal.

### 3.3. Histological Examination of the Cochlea

**Figure 5:**
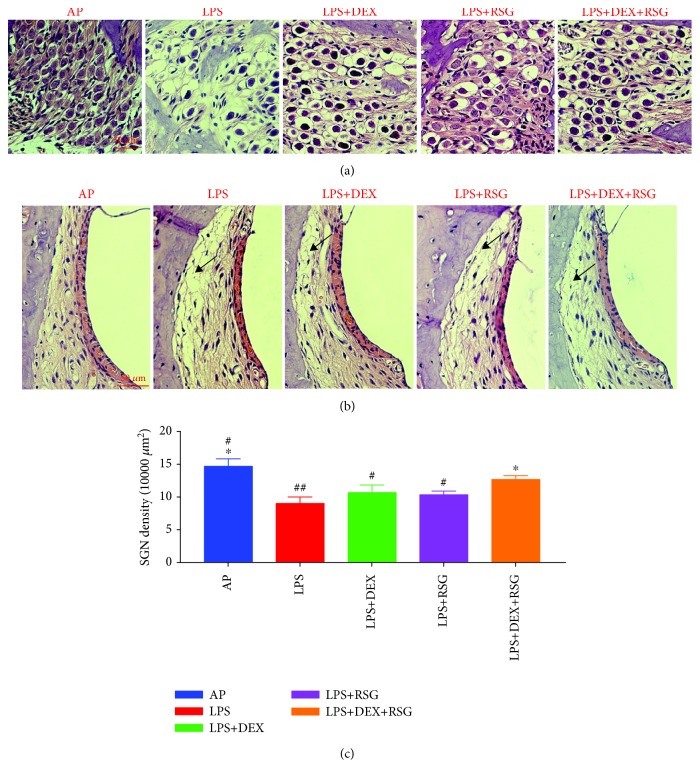
(a) Pathological observation of the spiral ganglion of pigs in each group. Scale bars = 50 *μ*m; (b) pathological observation of the stria vascularis in each group. Spiral ligament was sparse and disordered (black arrows). Scale bars = 50 *μ*m; (c) the bar graphs show the density of spiral ganglion cells in each group. Data are shown as means ± SD from different experiments, ^#^*p* < 0.05 vs. LPS+DEX+RSG group; ^##^*p* < 0.01 vs. LPS+DEX+RSG group; ^∗^*p* < 0.01 vs. LPS group.

The pathological observation of the cochlea in each group is shown in Figure 5. The HE stain uniformly stained the spiral ligaments in the AP group, and there was no gap within the cochlear lateral wall. The stria vascularis was normal in shape, with no rupture or erythrocyte exudation. The spiral ganglions were well arranged, and the nuclear/plasma ration was normal. In the LPS group, spiral ligaments were sparse and their structure was disordered. The spiral ganglion showed vacuolar degeneration and decreased nuclear/plasma ratio. These observations indicate that LPS destroys the inner ear morphology. However, the morphological damage was slightly decreased in the LPS+DEX and LPS+RSG groups, but some changes such as loosened spiral ligament and decreased nuclear/plasma ratio were still observed. The morphological recovery in the LPS+DEX+RSG group was the most obvious. The morphology of the stria vascularis and spiral ligament was normal, and there was no obvious gap between them and the lateral wall of the cochlea. The spiral ganglion/nucleoplast ration was also improved. The density of spiral ganglion cells in the LPS group was significantly lower than that in the AP group (*p* < 0.01). The LPS+DEX and LPS+RSG groups had no significant increase compared to the LPS group (*p* > 0.05), but the LPS+DEX+RSG group showed a significant increase compared to the LPS group (*p* < 0.01). Although the density of spiral ganglion cells in the LPS+DEX+RSG group was significantly lower than that in the AP group (*p* < 0.05), it was significantly higher than that in the LPS+DEX group or LPS+RSG group (*p* < 0.05). This indicates a combination of DEX, and RSG has a stronger protective effect than each alone.

### 3.4. Western Blot Analysis of MKP-1 and Related Proteins

**Figure 6:**
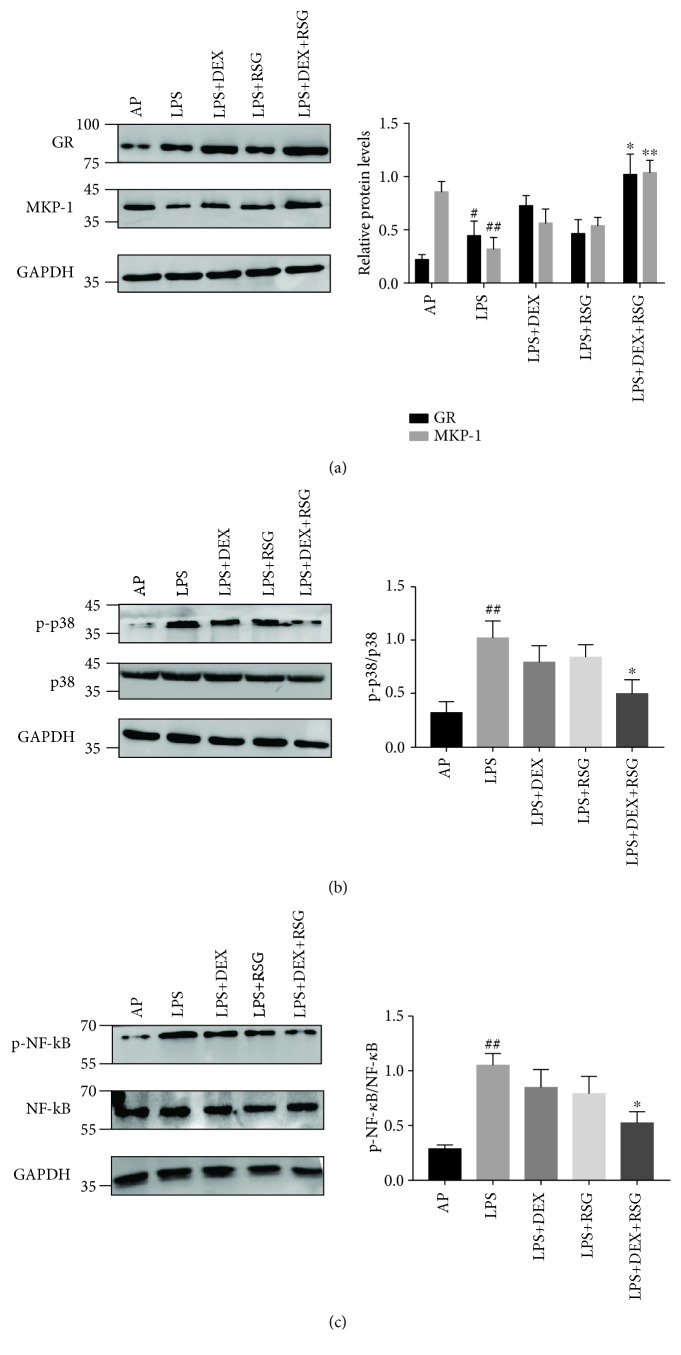
The protein expression of (a) MKP-1 and GR, (b) p38 and p-p38, and (c) NF-*κ*B and p-NF-*κ*B p65 was determined by western blot analysis. Bar graphs show the quantification of the indicated proteins. Data are shown as means ± SD from at least three independent experiments, ^#^*p* < 0.05 vs. AP group; ^##^*p* < 0.01 vs. AP group; ^∗^*p* < 0.05 vs. LPS+DEX group; ^∗∗^*p* < 0.01 vs. LPS+DEX group.

The expression of MKP-1, GR, p38, p-p38, NF-*κ*B p65, and p-NF-*κ*B p65 was determined by western blot analysis (Figure 6). The expression of MKP-1 in the LPS group was significantly lower than that in the AP group (*p* < 0.01). MKP-1 expression was not significantly higher in the LPS+DEX or LPS+RSG group compared to the LPS group (*p* > 0.05) but was significantly higher in the LPS+DEX+RSG group than in the LPS+DEX group (*p* < 0.01). Also, GR expression in the LPS group was significantly higher than that in the AP group (*p* < 0.05) and also significantly higher in the LPS+DEX+RSG group than the LPS+DEX group (*p* < 0.05). Additionally, the expression of p-p38/p-NF-*κ*B p65 in the LPS group was significantly higher than that in the AP group (*p* < 0.05), while it was significantly lower in the LPS+DEX group (*p* < 0.05) compared to the LPS+DEX+RSG group. In addition, there was no significant decrease in the LPS+DEX and LPS+RSG groups when compared to the LPS group (*p* > 0.05). These results suggest that a combination of DEX and RSG can significantly increase the expression of MKP-1 and GR. Moreover, lesions induced by LPS can be alleviated by inhibiting the activation of p38/p-NF-*κ*B.

## 4. Discussion

In this study, we investigated the role of MKP-1 and RSG in glucocorticoid resistance using a guinea pig model of LPS-induced SSHL. We demonstrated for the first time that MKP-1 protects inner ear tissues from inflammation-induced morphological damage and dysfunction by inhibiting the activation of p38 MAPK and NF-*κ*B. Our results also show that the synergy between RSG and DEX can increase the MKP-1 expression and reduce glucocorticoid resistance.

Although glucocorticoids are widely clinically used, a considerable number of patients show resistance to glucocorticoid therapy. These patients often have a high risk of poor prognosis not only due to the primary disease itself but also the adverse reactions resulting from the long-term use of glucocorticoids. Their visit and hospitalization rates are often far higher than those of other patients, thus increasing the economic burden of these individuals and the society. When glucocorticoids enter the plasmalemma and combine with GR, they can induce the rearrangement of the GR complex to facilitate its entry into the nucleus, for positive or negative regulation of gene transcription [21]. Multiple factors are involved in the molecular mechanism of glucocorticoid resistance [22, 23]. In recent years, it has been found that polymorphisms of the GR reduces its affinity to ligands, leading to glucocorticoid resistance [24]. Another study reported that glucocorticoid resistance is related to defects in GR expression and elevated expression of proinflammatory transcription factors [25]. Kojika et al. found that changes in HSP90 and HSP70 may be related to decreased glucocorticoid sensitivity. Abnormal HSP90 and HSP70 protein levels were successfully detected in two glucocorticoid-resistant human leukemia cell lines [26]. The increased expression of NF-*κ*B in the peripheral blood mononuclear cells of asthmatic patients was found to be negatively correlated with glucocorticoid responsiveness. The possible underlying mechanism is antagonism between NF-*κ*B and GR, which reduces glucocorticoid sensitivity [27].

Recently, Irusen et al. found that p38 MAPK activation can induce GR phosphorylation and function impairment [28]. In addition, p38 MAPK activation can increase the phosphorylation of the NF-*κ*B p65 subunit [29]. MKP-1 serves as an important regulator of the innate immune response by deactivating MAP kinases and NF-*κ*B [9–13]. Goleva et al. found that glucocorticoid resistance is associated with reduced induction of MKP-1, resulting in persistent p38 MAPK activation in peripheral blood mononuclear cells. Also, Bhavsar et al. reported that DEX could not induce the expression of MKP-1 in patients with severe (steroid-resistant) asthma [15]. Under the influence of inflammatory factors, MKP-1 knockout mice experienced increased levels of cytokines and chemical factors, increased neutrophil infiltration, and more severe organ damage than wild-type mice [30]. In addition, inflammation and oxidative stress also lead to SSHL. In the present study, we found that MKP-1 is expressed in inner and outer hair cells, supporting cells, and spiral ganglion cells in the cochlea. This was mainly observed in the cytoplasm and to some extent in the nucleus.

RSG is mainly used as an insulin sensitizer for the treatment of diabetes. Recent studies on peroxisome proliferative-activated receptor (PPAR) gamma have shown that RSG has anti-inflammatory and antioxidative effects [31–33]. Also, Tai et al. found that RSG could inhibit the proliferation and metastasis of non-small-cell lung cancer *in vivo* and *in vitro* via induction of MKP-1 [16]. RSG can also increase MKP-1 expression, which inhibits cell invasion in human glioma cells [34].

The anti-inflammatory effect of glucocorticoids after binding with GR is mainly achieved by enhancing the transcription of anti-inflammatory or inflammatory genes. MKP-1 is an important anti-inflammatory protein transcribed by glucocorticoids [35]. In this, as in a previous study, we found that the hearing function of guinea pigs with SSHL did not significantly improve after glucocorticoid treatment, which indicates glucocorticoid resistance or insensitivity [5]. Also, the threshold shift in the LPS+DEX+RSG group was the lowest, indicating MKP-1 could achieve its anti-inflammatory effect by inhibiting the activity of p38 MAPK in the inner ear. Further, the MKP-1 expression in the LPS+DEX+RSG group was higher than that in the LPS+DEX and LPS+RSG groups. DEX alone could not effectively increase the expression of MKP-1 in SSHL, which resulted in uncontrollable inflammation. Additionally, during LPS stimulation, GR expression was upregulated as a feedback effector, but its upregulation was more significant in the LPS+DEX+RSG group than in the LPS+DEX group. The LPS+DEX+RSG group had the best anti-inflammatory effect. Further, RSG exhibited anti-inflammatory effects, resulting in the restoration of decreased GR levels and impaired function caused by inflammation. Thus, when RSG is combined with DEX, the expression of MKP-1 can be enhanced. Therefore, RSG may play an important role in improving glucocorticoid resistance in SSHL and other inner ear disorders, owing to its ability of lower blood glucose levels, control inflammation, and eliminate toxins and side effects. It is therefore a potential new treatment option for such diseases.

There are certain limitations to our study. First, the guinea pig model of LPS-induced SSHL does not completely represent the clinical pathogenesis of SSHL. Further research should be performed using animal models more suitable for SSHL studies and clinical trials. Secondly, this is the first study to use the PPAR-*γ* agonist RSG for the treatment of an inner ear disease, the structure and function of PPAR-*γ* are very complex, and there are many unknown aspects to be explored.

We will investigate the route and administration time of RSG in future experiments.

## 5. Conclusion

In summary, MKP-1 is a key factor for the anti-inflammatory effect of hormones in inner ear tissues. The mechanism of glucocorticoid resistance in the guinea pig model of LPS-induced SSHL may be associated with reduced levels of MKP-1 induction, resulting in persistent MAPK activation in the inner ear. The combined use of RSG and glucocorticoids provides a new treatment option for glucocorticoid resistance in SSHL.

## Data Availability

To anyone who needs dates, please contact the corresponding authors.
